# Mechanisms connecting square dance to sleep quality among middle-aged and older Chinese females: serial mediation roles of social support and depressive symptoms

**DOI:** 10.3389/fpubh.2023.1307596

**Published:** 2023-11-23

**Authors:** Jun Wu, Yong Yu, Keke Qin, Zhiwen Ou

**Affiliations:** ^1^School of Music and Dance, Shaoyang University, Shaoyang, Hunan, China; ^2^School of Politics and Public Administration, Guangxi Normal University, Guilin, Guangxi, China; ^3^School of Marxism, Shaoyang University, Shaoyang, Hunan, China

**Keywords:** square dance, sleep quality, social support, depressive symptoms, middle-aged and older Chinese women

## Abstract

**Background:**

Square dance is gaining increasing popularity among middle-aged and older Chinese women who are also at high risk of sleep disturbance. Although previous studies have shown exercise could improve sleep quality, the association between square dance and sleep quality remains to be discussed, and even less is known about the potential mechanism underlying this association.

**Purpose:**

This study aims to investigate the relationship between square dance and sleep quality and test if social support and depressive symptoms together play a serial mediating role in the influence of square dance on sleep quality.

**Methods:**

A cross-sectional study was conducted among 549 middle-aged and older Chinese females from September to December 2020 in Shao Yang City, Hunan Province of China, with ethics approval granted (SYU [2020]002). Square dance involvement was assessed by three questions about the time participants spent in square dance. Social support, depressive symptoms, and sleep quality were measured using the Pittsburgh Sleep Quality Index (PSQI), Social Support Self-Rating Scale (SSRS), and 9-item Patient Health Questionnaire (PHQ-9), respectively. The serial mediation model was analyzed by the bootstrapping method to assess whether social support and depressive symptoms mediate the relationship between square dance and sleep quality.

**Results:**

Two-thirds of the participants had high involvement in square dance and most reported a moderate and high level of social support (98.54%). The prevalence of depressive symptoms and sleep disturbance was 19.49 and 26.78%, respectively. The serial mediation model showed a significant association between square dance and sleep quality, which was fully mediated by social support and depressive symptoms in a serial model (total effect c = −0.114, 95%CI = −0.227 to −0.001; direct effect c’ = −0.036, 95% CI = −0.138 to 0.065; total indirect effect ab = −0.077, 95% CI = -0.139 to-0.016).

**Conclusion:**

Our study extends the understanding of how square dance is associated with sleep quality through the serial mediating roles of social support and depressive symptoms. It provides crucial implications for developing square dance interventions to improve sleep quality among middle-aged and older Chinese females.

## 1 Introduction

Square dance, also known as grannies’ dance, is a spontaneous group leisure activity consisting of music, a dance leader, and a group of dancers (1, 2). The square dance usually occurs in public spaces such as squares, parks, playgrounds, courtyards, or recreation areas during the night when people are at leisure (3). The forms of square dance are diverse and easy to learn, ranging from gymnastic exercises to disco, folk dance, and modern dance (4). As a physical exercise that combines fitness and entertainment, square dance has gained increasing popularity in China, with an estimated 100 million everyday practitioners (4). It has significantly contributed to China’s increased prevalence of physical activity to prevent non-communicable chronic diseases (NCDs) and improve health (5, 6). Square dance is prevalent among middle-aged and older Chinese women who are in a life stage of facing multiple challenges, such as deterioration of physiological function after menopause, loss of socioeconomic status after retirement, social isolation, and bereavement and has been recommended as an effective tool to help them go through these life challenges (7).

The beneficial roles of square dance in promoting human health have been well-documented, with abundant evidence showing its health-promoting effects in terms of physical, mental, and social wellbeing (5). As a moderate-intensity exercise, square dance has been shown to significantly reduce body mass index and decrease the risk of obesity-related NCDs such as coronary heart disease, hypertension, diabetes, and stroke (8). It also enhances muscle strength, promotes body balance, and strengthens immunity functioning (5, 9). Regarding mental benefits, both observational and interventional studies have demonstrated that square dance could significantly reduce depressive symptoms, improve quality of life, and promote subjective wellbeing (10, 11). As for social benefits, square dance aligns with the Chinese collectivist culture and dramatically enhances social interaction (12), which helps alleviate social isolation and loneliness in the context of the rapidly aging population (13). Research indicated that square dance could significantly expand participants’ social network, increase social support, foster self-actualization, improve intergenerational communication, and enhance family cohesion, contributing to a healthier social life (14, 15).

Sleep disturbance, characterized by persistent inability to fall asleep or maintain sleep, is a significant public health problem affecting over 10% of the worldwide population (16, 17). The prevalence of sleep disturbance increases with age and is especially common among middle-aged and older women during and after the menopause transition, with a reported prevalence ranging from 40 to 60% (18, 19). Sleep disturbance has deleterious effects on physical and mental health, leading to impaired metabolic, endocrine, and immune systems, increased risk of psychological distress, and low levels of quality of life (20). Previous studies have demonstrated multilevel risk factors of sleep disturbance, including demographic (e.g., older age and female), behavioral (e.g., lower physical activity levels), psychological (e.g., depressive symptoms), and social factors (e.g., social isolation and lack of social support) (21). Specifically, lack of physical activity has been identified as the most modifiable factor that affects sleep quality among middle-aged and older people, with abundant evidence showing significant correlations between physical inactivity with depression, cognition, the aging process, and sleep disturbance (22–25). A recent literature review and meta-analysis also demonstrated significant positive effects of exercise training on sleep quality in middle-aged and older adults (20). However, few studies have focused on the effect of square dance on sleep quality among middle-aged and older Chinese females, and even less is known about the mechanism connecting square dance to sleep quality. Despite solid evidence showing that low social support and depressive symptoms were risk factors for sleep disturbance, and that square dance could improve social support and decrease depressive symptoms, no study has ever connected square dance and sleep quality through the mediation roles of social support and depressive symptoms.

The current study aims to fill those gaps by studying the mechanisms by which square dance impacts sleep quality in middle-aged and older Chinese females. Based on previous research regarding the significant associations among square dance, social support, depressive symptoms, and sleep disturbance, this study hypothesized that square dance indirectly reduces the risk of sleep disturbance through social support and depressive symptoms in two separate serial mediation models. Serially, we hypothesized that higher involvement in square dance would be associated with higher levels of social support and, in turn, fewer depressive symptoms, thus decreasing the risk of sleep disturbance. Therefore, we proposed the following hypotheses.

*Hypothesis 1.* There is an association between square dance and sleep disturbance (total effect c);

*Hypothesis 2.* There is a specific indirect effect of square dance on sleep disturbance through social support (indirect effect a1b1);

*Hypothesis 3.* There is a specific indirect effect of square dance on sleep disturbance through depressive symptoms (indirect effect a2b2);

*Hypothesis 4.* There is a serial multiple mediation effect of square dance on sleep disturbance through social support and then through depressive symptoms (serial or cascading indirect effect a1a3b2).

## 2 Methods

### 2.1 Participants

Data were collected from a cross-sectional questionnaire survey conducted from September to December 2020 in Shaoyang City, Hunan Province of China. The target population was middle-aged and older females who regularly participated in the square dance between 18:00–21:00 in Shaoyang City. The sample size was calculated according to the form for cross-sectional study: *n* = z (2) P (1− P)/E^2^, where P (the prevalence of sleep disturbance) was estimated at 40% based on past studies (18, 19), Z was set as 1.96 at a confidence interval of 95%, and the allowable error was set as 5%. Considering a rejection or loss-to-follow-up rate of 20%, we expanded our final sample size to 461. In order to get a sample that is as representative of all rural residents of Shaoyang City as possible, a two-stage random cluster sampling method was adopted to recruit participants. In the first stage, one district (Daxiang District) was randomly selected from 3 districts of Shaoyang City. In the second stage, 40 communities and villages in Daxiang District were randomly selected out of 97 communities and villages. Inclusion criteria were: (1) aged ≥40, (2) female, (3) living in the community for more than 3 months, (4) performing regular square dance for more than 3 months with fewer than 7 days of non-performing gap, (5) with normal cognitive ability and literacy to complete questionnaire survey, (6) agreeing to participate in the study with informed consent. Exclusion criteria were: (1) not living in the community during the research period, (2) having difficulty in communication due to severe physical or mental illness. A final sample of 549 females was recruited, which satisfies our sample size requirement.

### 2.2 Procedure

Ethics approval was granted by the Ethics Review Committee of Shaoyang College (SYU [2020]002). Data were collected by a research team composed of 10 college students from the Music and Dance Academy of Shaoyang College. The research team received unified and standardized training combining lecture and role-play practice on questionnaire interviews prior to the formal investigation. The research team approached the participants in each square during the night when they were performing square dance and explained the purpose and procedure of the study in detail to each interested participant. After providing written informed consent, the eligible participants were invited to complete a battery of questionnaires. Considering most participants were middle-aged and older people who may have difficulty reading and filling in the questionnaires by themselves, the research team conducted face-to-face interviews with the participants and then filled in the questionnaires for the participants. The answers were then checked by a quality control person on the spot to ensure integrity, accuracy, and consistency.

### 2.3 Measures

#### 2.3.1 Square dance involvement

Square dance involvement was assessed by three questions asking about the time participants spent in square dance. The first question asked how many years participants had been involved with square dance, with optional answers ranging from 1 (less than 1 year) to 5 (more than 10 years). The second question asked how often participants performed square dance, with optional answers ranging from 1 (less than once a month) to 5 (almost every day). The third question asked about the average length of each square dance session, with optional answers ranging from 1 (less than 10 min) to 5 (more than 60 min). A total involvement score was calculated by summing up the three question scores and ranged from 3 to 15, with a higher score indicating higher involvement in square dance.

#### 2.3.2 Sleep quality

Sleep quality was measured using the Pittsburgh Sleep Quality Index (PSQI), which is the most commonly used standardized self-administered scale to assess sleep quality and sleep disturbance over the previous month (26). Ever since its development in 1989, the PSQI has been translated into over 50 languages and validated in both clinical and non-clinical populations across cultures (27). It has been widely accepted as a useful instrument for assessing sleep disturbance associated with psychological distress. The PSQI includes 19 items under seven clinically derived domains of sleep difficulties: sleep quality, sleep latency, sleep duration, habitual sleep efficiency, sleep disturbances, use of sleeping medications, and daytime dysfunction. Each domain has a range of 0–3 points, with 0 points indicating no difficulty and 3 points indicating severe difficulty. The total score ranges from 0 to 21, with a higher score representing higher sleep difficulty. The PQSI was first translated into Chinese by Liu et al. (28) in 1996 and has shown good reliability and validity in Chinese populations (29, 30). A PSQI score greater than seven was deemed sleep disturbance in the Chinese population (28). In the current study, the PSQI showed good internal consistency with a Cronbach α of 0.82.

#### 2.3.3 Social support

Social support was measured using the Social Support Self-Rating Scale (SSRS) developed by Xiao (31) to assess multiple dimensions of social support and is one of the most commonly used social support scales in China. It consists of 10 items under three dimensions: subjective support (4 items), objective support (3 items), and support utilization (3 items). Items 1–4 and 8–10 are scored from 1 to 4 points. Item 5 includes four support sources, and each source is scored from 1–4 points, leading to a total score of 4–20 points. Items 6 and 7 are scored from 0 to 9 points depending on the nine sources of support. The total support score ranges from 12 to 66, with a higher score indicating a higher level of social support, which is further classified into three categories: low (≤22), moderate (23–44), and high (≥45) levels of support. In the current study, the SSRS showed good internal consistency with a Cronbach α of 0.75.

#### 2.3.4 Depressive symptoms

Depressive symptoms were measured using the 9-item Patient Health Questionnaire (PHQ-9) (32) to assess nine depressive symptoms during the past 2 weeks. Each item is rated on a 4-point Likert scale from 0 (not at all) to 3 (nearly every day). The total score ranges from 0 to 27, with a higher score implying more depressive symptoms, cutoffs of 5, 10, 15, 20 for each level of depression, and a cutoff point of 10 differentiating between depression and non-depression (33, 34). The PHQ-9 showed good reliability with a Cronbach’s alpha of 0.89 in the original study (32). The PHQ-9 was first translated into Chinese by Yeung et al. (35) in 2008 and proved to be a reliable and valid measure of depression. In the current study, the PHQ-9 showed good internal consistency, with a Cronbach’s alpha of 0.90.

#### 2.3.5 Covariates

To account for observable potential confounding effects of sociodemographic variables, we collected the following variables as covariates in the fully adjusted models: age, race (ref: Han), education (ref: primary and below), employment (ref: not employed), income (ref:<4,000), marital status (ref: not married), illness (ref: no), hospitalization (ref: no), and medical insurance (ref: no).

### 2.4 Statistical analyses

Descriptive and correlation analyses were conducted using Stata v.15 (36) for all variables. Continuous variables were presented by means and standard deviations while categorical variables were described by frequencies and proportions. Pearson’s Product Moment correlation analysis was performed to examine the associations among the four variables: square dance involvement, social support, depressive symptoms, and sleep disturbance. A correlation coefficient of 0.80 and above indicates multi-collinearity (37).

**Figure 1:**
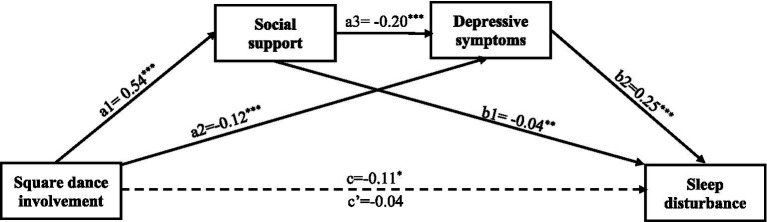
Schematic of an (in)direct effects serial mediation model for the relationship of square dance involvement and sleep disturbance with social support and depressive symptoms. a1 = direct effect of square dance involvement on social support; a2 = direct effect of square dance involvement on depressive symptoms; a3 = direct effect of social support on depressive symptoms; b1 = direct effect of social support on sleep disturbance; b2 = direct effect of depressive symptoms on sleep disturbance; c = total effect of square dance involvement on sleep disturbance not accounting for social support and depressive symptoms; c’ = direct effect of square dance involvement on sleep disturbance accounting for social support and depressive symptoms. For specific total indirect effect results, refer to Table 3. *p < 0.05, **p < 0.01, ***p < 0.001.

The serial mediation analysis was carried out using SPSS PROCESS v.4.3 macro (Model 6) (38). A serial mediation model proposes that one mediator affects another mediator, such that square dance involvement (X) could increase social support (mediator 1), which could decrease depressive symptoms (mediator 2), thus alleviating sleeping disturbance (Y). The total effect of X on Y is denoted as c, which includes one direct effect represented by path c′ and three indirect effects: (1) through social support alone (a1b1), (2) through depressive symptoms alone (a2b2), and (3) through social support and depressive symptoms in successive order (a1a3b2) (this is shown in Figure 1). In our analyses, we controlled for age, race, education, employment, income, marital status, illness, hospitalization, and medical insurance. A bootstrapping method based on 5,000 samples was used to determine the significance of mediators, assessing indirect, direct, and total effects of square dance involvement on sleep disturbance. For this model, an effect was considered significant if its 95% bootstrap confidence interval did not include zero (38).

## 3 Results

### 3.1 Descriptive statistics

Table 1 displays the sociodemographic characteristics of participants and descriptive statistics of all study variables. Participants had a mean age of 48.16 years (SD = 7.78). Most participants were of Han nationality (94.35%), employed (61.02%), and married (88.34%). The majority of participants had a middle and high school education (57.38%), a monthly income of ≤4,000 yuan (68.12%), and medical insurance (81.06%). Forty-one (7.47%) reported having a physical illness, and 90 (16.39%) had been hospitalized.

**Table 1:** Sample characteristics (*n* = 549).

Variable		Mean (SD)/N (%)
Demographical characteristics		
Age	M (SD)	48.16 (7.78)
Race	Han	518 (94.35)
	Non-Han	31 (5.65)
Education	Primary school and below	103 (18.76)
	Middle and high school	315 (57.38)
	College and above	131 (23.86)
Employment	Not employed	214 (38.98)
	Employed	335 (61.02)
Monthly income	≤4,000	374 (68.12)
	>4,000	175 (31.88)
Marital status	Not married	64 (11.66)
	Married	485 (88.34)
Physical illness	No	508 (92.53)
	Yes	41 (7.47)
Hospitalization	No	459 (83.61)
	Yes	90 (16.39)
Medical insurance	No	104 (18.94)
	Yes	445 (81.06)
Square dance involvement	M (SD)	9.19 (2.14)
Square dance length (years)	<1	152 (27.69)
	1–3	210 (38.25)
	3–5	121 (22.04)
	5–10	44 (8.01)
	>10	22 (4.01)
Square dance frequency	Less than once a month	20 (3.64)
	2–3 times per month	33 (6.01)
	1–2 times per week	123 (22.40)
	3–5 times per week	267 (48.63)
	Almost every day	106 (19.31)
Square dance session (minutes)	<10	31 (5.65)
	11–20	94 (17.12)
	21–30	203 (36.98)
	31–60	163 (29.69)
	>60	58 (10.56)
Social support	M (SD)	41.78 (7.54)
	Low	8 (1.46)
	Moderate	337 (61.38)
	High	204 (37.16)
Depressive symptoms	M (SD)	5.40 (1.93)
	No	442 (80.51)
	Yes	107 (19.49)
Sleep disturbance	M (SD)	5.94 (2.78)
	No	402 (73.22)
	Yes	147 (26.78)

The mean total score of square dance involvement was 9.19 ± 2.14. Most participants had been involved in square dance for less than 3 years (65.94%). Most went to square dance at least three times per week (67.94%) for 20–60 min each time (66.67%). The mean score of SSRS was 41.78 ± 7.54. Most participants reported having a moderate (61.38%) or high (37.16%) level of social support. The prevalence of depressive symptoms was 19.49% (*n* = 107) with a mean PHQ-9 score of 5.40 ± 1.93. Prevalence of depression at a mild level (PHQ-9 ≥ 5, PHQ-9 < 10), moderate level (PHQ-9 ≥ 10, PHQ-9 < 15), and severe level (PHQ-9 ≥ 15) was 30.05%, 15.85%, and 3.65%, respectively. The prevalence of sleep disturbance was 26.78% (*n* = 147), with a mean global PSQI score of 5.94 ± 2.78.

### 3.2 Correlations on the key variables

Table 2 shows the correlation coefficients among the key variables by Pearson’s Product Moment correlation analysis. All the correlations were statistically significant in the predicted directions and did not exceed the recommended cut-off for multi-collinearity (*r* > 0.80) (37). Specifically, square dance involvement was positively correlated with social support (*r* = 0.35, *p* < 0.001) and negatively correlated with depressive symptoms (*r* = −0.32, *p* = 0.007) and sleep disturbance (*r* = −0.29, *p* = 0.027). Sleep disturbance was positively correlated with depressive symptoms (*r* = 0.46, *p* < 0.001) and negatively correlated with social support (*r* = −0.27, *p* < 0.001). Both mediators, social support and depressive symptoms, were negatively associated with each other (*r* = −0.31, *p* < 0.001).

**Table 2:** Pearson’s correlations among study variables.

Study variables	1	2	3	4
1. Square dance involvement	1			
2. Social support	0.35***	1		
3. Depressive symptoms	−0.32**	−0.31***	1	
4. Sleep disturbance	−0.29*	−0.27***	0.46***	1

*p < 0.05, **p < 0.01, ***p < 0.001.

### 3.3 Serial-mediation model analysis

Figure 1 and Table 3 show the total, direct, and indirect effects of square dance involvement and sleep disturbance through social support and depressive symptoms.

**Table 3:** Total, direct, and indirect effects of square dance involvement on sleep disturbance.

Path	B	SE	LLCI	ULCI
Total effect (c)	−0.1136	0.0576	−0.2268	−0.0005
Direct effect (c’)	−0.0364	0.0512	−0.1375	0.0648
Total indirect effects (ab)	−0.0773	0.0314	−0.1390	−0.0162
Square dance → social support → sleep disturbance (a1b1)	−0.0216	0.0144	−0.0458	−0.0038
Square dance → depressive symptoms → sleep disturbance (a2b2)	−0.0296	0.0267	−0.0841	−0.0208
Square dance → social support → depressive symptoms → sleep disturbance (a1a3b2)	−0.0261	0.0084	−0.0437	−0.0100

Number of bootstrap samples for bias-corrected bootstrap CIs: 5,000. CI, confidence interval; LLCI, low limit confidence interval; ULCI, upper limit confidence interval.

Confirming hypothesis 1, the association between square dance involvement and sleep disturbance after controlling for covariates was statistically significant (total effect c = −0.114, 95%CI = −0.227 to −0.001), indicating higher square dance involvement was associated with less sleep disturbance. The combined contribution of square dance involvement and covariates explained 14.2% of the total variance. However, the association between square dance involvement and sleep disturbance was not statistically significant when mediators (social support and depressive symptoms) were added (direct effect c’ = −0.036, 95% CI = −0.138 to 0.065), indicating a significant full mediation effect by the mediators. Square dance involvement was unrelated to sleep disturbance, independent of the effect of social support and depressive symptoms. The total indirect effect of square dance involvement on sleep disturbance was significant (ab = −0.077, 95% CI = −0.139 to −0.016).

Confirming hypothesis 2, The indirect effect of square dance involvement on sleep disturbance through social support was significant (a1b1 = −0.022, 95% CI: −0.046 to−0.004), indicating that those who had higher square dance involvement had higher social support (a1 = 0.54, *p* < 0.001), which in turn was associated with lower sleep disturbance (b1 = −0.04, *p* = 0.009). The indirect effect of square dance involvement on sleep disturbance through depression was significant (a2b2 = −0.030, 95% CI: −0.046 to−0.004), indicating that those who had higher square dance involvement had lower depressive symptoms (a2 = −0.12, *p* < 0.001), which in turn was associated with lower sleep disturbance (b2 = 0.25, *p* < 0.001), confirming hypothesis 3. Finally, the indirect effect of square dance involvement on sleep disturbance through social support and depressive symptoms serially was significant (a1a3b2 = −0.026, 95% CI = −0.044 to −0.010). Thus, hypothesis 4 was also confirmed. Those with higher square dance involvement had a higher level of social support (a1 = 0.54, *p* < 0.001), which was associated with fewer depressive symptoms (a3 = −0.20, *p* < 0.001), and which, in turn, was associated with less sleep disturbance (b2 = 0.25, *p* < 0.001). Among the three mediation paths, depressive symptoms account for the largest proportion of the mediation effect (38%), followed by social support and depressive symptoms serially (34%) and social support (28%).

## 4 Discussion

### 4.1 Summary of the findings

Square dance is a popular leisure activity for middle-aged and older Chinese women. It has been documented to produce physical, mental, and social benefits to help them overcome life challenges. Sleep disturbance is common among middle-aged and older women and is affected by multilevel risk factors, including demographic, behavioral, psychological, and social factors. Although physical activity has been shown to improve sleep disturbance, few studies have exclusively investigated the impact of square dance on sleep disturbance among middle-aged and older Chinese women. Although both square dance and sleep disturbance were associated with social support and depressive symptoms, no study has connected square dance with sleep disturbance through social support and depressive symptoms. The main aim of our study was to test if there was a relationship between square dance and sleep disturbance and if social support and depressive symptoms together play a serial mediating role in the influence of square dance on sleep disturbance among middle-aged and older Chinese women.

To the best of our knowledge, this was the first study to investigate the relationship between the variables using the serial mediation model. As expected, our results showed that square dance was significantly associated with sleep disturbance, and the effect was fully mediated by social support and depressive symptoms. In addition, social support and depressive symptoms played a serial mediating role in the influence of square dance on sleep disturbance. Our findings provide significant insights into square dance’s beneficial role and underlying mechanism in improving sleep quality among middle-aged and older Chinese women.

### 4.2 Square dance and sleep disturbance

The square dance participant profile was consistent with a 48-year-old married, employed woman with a middle-level education who went to square dance at least three times per week for 20–60 min each time in the past 3 years. The prevalence of sleep disturbance assessed by the PSQI in the current study was 26.78%, much lower than the reported 40–60% among middle-aged and older females in previous studies (18, 19). This indicates that participation in square dance could improve sleep quality, which is further supported by the statistically significant association between square dance involvement and sleep quality score, showing a significant total effect. Our findings further confirm hypothesis 1 and validate the beneficial role of square dance in mitigating sleep disturbance and promoting sleep quality. This result is consistent with a previous literature review demonstrating that exercise could improve sleep quality in middle-aged and older adults (20). It indicates that square dance can be recommended as an alternative to other exercise programs to improve sleep. One finding suggests that the development and popularization of square dance intervention programs may be a cost-effective way to improve sleep quality among middle-aged and older females. As square dance has shown significant positive effects in relieving psychological distress and improving quality of life in previous interventional studies (10), future interventional studies are warranted to test its effect on sleep quality further.

### 4.3 The serial mediating effect of social support and depressive symptoms

Our study showed a surprisingly high level of social support among square dance participants, with 98.54% of participants reporting moderate and high levels of social support, implying that square dance could potentially improve social support. The prevalence of depressive symptoms in our study was 19.49%, which was much lower than the reported 38.37% among middle-aged and older Chinese women in a national study (39). This may be explained by the high social support level of participants who performed square dance regularly, as social support is a well-established protective factor to combat depressive symptoms. These findings were further echoed in the serial mediation analysis showing a full mediation effect of social support and depressive symptoms. The direct effect of square dance on sleep disturbance was non-significant after adding the two mediators: social support and depressive symptoms. Square dance does not directly improve sleep quality but indirectly through improved social support and decreased depressive symptoms, both separately and serially, further confirming hypotheses 2–4.

Square dance has been shown to promote social integration by providing opportunities for participants to know more people and develop friendships, strengthening both objective and subjective social support (12). Social support is a well-demonstrated protective factor in reducing and preventing depressive symptoms, which has been widely and consistently reported in numerous studies (40). Higher levels of social support are also associated with improved sleep quality, as evidenced by a recent meta-analysis (41). In addition, our study showed that depressive symptoms were significantly related to sleep disturbance, which was in line with previous literature showing a high correlation between depression and sleep disturbance (42). The associations between square dance, social support, depressive symptoms, and sleep quality may also be explained by several physiological mechanisms. Square dance may promote mental health and sleep by altering the body’s pro-inflammatory factors, sleep–wake rhythms, and 5-HT levels (24, 25, 43). Square dance may also induce the secretion of dopamine, which increases the individual’s experience of pleasure and reduces stress and negative emotions (24, 25, 43). In summary, our study was consistent with previous studies and showed that square dance could increase social support, which could reduce depressive symptoms and, in turn, decrease sleep disturbance. These findings have important implications for designing and implementing square dance interventions to improve sleep quality among middle-aged and older Chinese females, with a particular focus on improving social support and decreasing depressive symptoms.

### 4.4 Limitations

This study has several limitations, which may motivate future research. First, the cross-sectional study design may preclude causal inference among the study variables, which needs to be further tested in future longitudinal study designs. Second, square dance involvement was assessed by three self-designed questions based on frequency and duration, which may not fully capture other aspects of square dance involvement, such as the degree of satisfaction, happiness, and passion. The development of a comprehensive and psychometrically sound scale to measure square dance involvement is warranted in future studies. Third, study participants were selected from one district of Shaoyang City, which may not represent participants from other districts or other cities of China. Future studies may consider recruiting a national-level sample to get a complete picture and compare the geographic differences in square dance and sleep disturbance. Fourth, we did not include many other factors that may affect our results, such as medication, drinking, smoking, BMI, and chronic illness, which have been shown to significantly affect sleep quality and depression by accumulating evidence (44–47). In addition, we did not collect further information about participants’ social networks (such as the number of friends/family members) and engagement in other activities (such as sports, arts, and social clubs), which may also affect their social support. Future studies should consider adding these factors for a more comprehensive understanding of anthropometric and lifestyle characteristics’ effects on the relationship between square dance and sleep disturbance. Specifically, the influence of short sleep on mental health is an important topic that warrants further studies, as recent publications have shown that short sleep is another emerging issue, and physical exercise could mitigate the influence of short sleep on cognition (43, 48).

## 5 Conclusion

In sum, this study confirms the significant association between square dance and sleep quality among middle-aged and older Chinese females, and this association was fully mediated by social support and depressive symptoms in a serial model. People with higher square dance involvement have higher levels of social support, which in turn, are associated with fewer depressive symptoms, thus leading to less sleep disturbance. Our study extends the understanding of how square dance is associated with sleep quality through the serial mediating roles of social support and depressive symptoms. These results provide important research and practical guidance in developing square dance interventions to improve the sleep quality of middle-aged and older Chinese females.

## Data availability statement

The raw data supporting the conclusions of this article will be made available by the authors, without undue reservation.

## Ethics statement

The studies involving humans were approved by the Institutional Review Committee of Shao Yang College (SYU[2020]002). The studies were conducted in accordance with the local legislation and institutional requirements. The participants provided their written informed consent to participate in this study.

## Author contributions

JW: Conceptualization, Data curation, Formal analysis, Investigation, Methodology, Project administration, Resources, Software, Visualization, Writing – original draft. YY: Conceptualization, Data curation, Funding acquisition, Investigation, Methodology, Project administration, Resources, Software, Supervision, Validation, Visualization, Writing – review & editing. KQ: Conceptualization, Data curation, Funding acquisition, Investigation, Methodology, Project administration, Resources, Software, Supervision, Validation, Visualization, Writing – review & editing. ZO: Conceptualization, Data curation, Investigation, Project administration, Resources, Software, Supervision, Validation, Visualization, Writing – review & editing.
